# A double amino-acid change in the HLA-A peptide-binding groove is associated with response to psychotropic treatment in patients with schizophrenia

**DOI:** 10.1038/tp.2015.97

**Published:** 2015-07-28

**Authors:** S Le Clerc, L Taing, G Fond, A Meary, P-M Llorca, O Blanc, P Beaune, K Rajagopal, S Jamain, R Tamouza, J-F Zagury, M Leboyer

**Affiliations:** 1 Équipe EA4627, Chaire de Bioinformatique, Conservatoire National des Arts et Métiers, Paris, France; 2 INSERM, U955, Psychiatrie Génétique, Créteil, France; 3 Université Paris-Est, Faculté de Médecine, Créteil, France; 4 AP-HP, DHU PePSY, Pôle de Psychiatrie, Hôpitaux Universitaires Henri Mondor, Créteil, France; 5 Fondation FondaMental, Créteil, France; 6 Service de Psychiatrie Adulte, Hôpital Gabriel Montpied, Clermont-Ferrand, France; 7 INSERM, U775, Centre de recherches Biomédicales, Université Paris Descartes, Paris, France; 8 Laboratoire Jean Dausset (LabEx Transplantex) et INSERM, U1160, Hôpital Saint Louis, Paris, France; 9 Université Paris Diderot, Sorbonne Paris-Cité, Paris, France

## Abstract

The choice of an efficient psychotropic treatment for patients with schizophrenia is a key issue to improve prognosis and quality of life and to decrease the related burden and costs. As for other complex disorders, response to drugs in schizophrenia is highly heterogeneous and the underlying molecular mechanisms of this diversity are still poorly understood. In a carefully followed-up cohort of schizophrenic patients prospectively treated with risperidone or olanzapine, we used a specially designed single-nucleotide polymorphism (SNP) array to perform a large-scale genomic analysis and identify genetic variants associated with response to psychotropic drugs. We found significant associations between response to treatment defined by the reduction in psychotic symptomatology 42 days after the beginning of treatment and SNPs located in the chromosome 6, which houses the human leukocyte antigen (HLA). After imputation of the conventional *HLA* class I and class II alleles, as well as the amino-acid variants, we observed a striking association between a better response to treatment and a double amino-acid variant at positions 62 and 66 of the peptide-binding groove of the HLA-A molecule. These results support the current notion that schizophrenia may have immune-inflammatory underpinnings and may contribute to pave the way for personalized treatments in schizophrenia.

## Introduction

Given the important prevalence, the severity and the high socio-economic burden of schizophrenia (SZ), pharmacogenomics has brought hopes of improvements, notably by helping predict response to drugs according to individuals' genetic make-up. SZ is a chronic and remitting disorder associated with progressive deterioration of cognitive and behavioral functioning with a worldwide lifetime prevalence of 1.4–4.6%.^1^ In UK, it has recently been estimated that SZ costs every year more than £11.8 billion (US$19.7 billion).^2^ Much of the cost is attributed to the fact that ~30–50% of SZ patients do not respond adequately to antipsychotic treatment leading to relapses and hospitalizations with considerable burden and socio-economic concerns.^3^ Even a modest improvement in treatment outcome would thus provide a great benefit by reducing burden and costs.

Although antipsychotic treatment has consistently and continuously been recommended as the standard therapeutic approach for SZ, there is total lack of relevant clinical predictors of their inter-individual responses despite intensive research efforts.^4^ Such predictors would be hence of major interest.

The mechanisms of action of the second-generation antipsychotics, such as olanzapine or risperidone, are still poorly understood as the vast majority of genetic studies on drug response have been essentially focused on loci implicated in monoamine pathways. However, in agreement with the role played by the catecholamine-O-methyl transferase enzyme both in the degradation of endogenous catecholamines and in the metabolism of some drugs including olanzapine and risperidone, differential responses to antipsychotic drugs have been associated with genetic variations lying in the catecholamine-O-methyl transferase gene.^5^ The TaqIA1 allele of the *DRD2* gene, the *DRD3* Ser9Gly and the *HTR2C*-759C/T polymorphisms are plausible biomarkers for response and/or adverse drug reactions.^6^

Since 2007, large-scale genome-wide association studies (GWAS) were proven to be a powerful tool to identify disease-related gene variants for many common human disorders and other traits. McClay *et al.*^7^ reported a GWAS for antipsychotic response and identified a single-nucleotide polymorphism (SNP) in an intergenic region on chromosome 4p15, while Ikeda et *al.*^8^ described an association between SNP in a gene encoding a component of phosphodiesterase (PDE) enzyme, namely *PDE7B* and response to risperidone.

Regarding plausible mechanisms, there is a large consensus that a significant proportion of patients with SZ exhibits features of chronic low-grade inflammation, evidenced by raised pro-inflammatory cytokine circulating levels, highlighted by the presence of circulating autoantibodies against brain receptors, altered activation of pro-inflammatory monocytes and anti-inflammatory T cells as well as by the prominent SZ-associated non-psychiatric autoimmune/metabolic comorbidities. Moreover, a number of GWAS repeatedly confirmed the pivotal implication of the human leukocyte antigens (HLAs) region on chromosome 6, a key regulatory region of the immune response, in SZ risk,^9, 10^ potentially reflecting the observed immune/inflammatory disturbances in affected patients.^9, 10^ These findings have again been very recently replicated within the largest consortium study on schizophrenia genetics, which detected the highest *P*-values in the MHC region (*P*<10^−^^30^).^9^ Interestingly, it has been observed that some of the antipsychotic drugs alleviate the immune-inflammatory stigma as evidenced by reduction in the peripheral levels of activated immune cells and pro-inflammatory markers in a proportion of SZ patients.^11^ Despite such contextual findings, to our knowledge, potential genetic association between genes involved in the immune system and response to antipsychotic treatment, have so far not been explored directly. In the present study, we performed a large-scale genomic study using a customized SNP array targeting functionally relevant genetic polymorphisms involved in the immune-inflammatory and pharmacogenetic pathways. Using this dedicated tool, we studied a cohort of 89 patients with SZ prospectively followed-up and characterized for their response to a single antipsychotic drug (olanzapine or risperidone).

## Materials and methods

### Ethics statement

Protocols and procedures were approved by the ethical review board (CCPPRB) of the Pitié-Salpêtrière Hospital in Paris. Written informed consent was obtained from all the subjects before study inclusion.

### Study design and population

We performed a non-interventional, multicentric prospective study. Patients were recruited in two university-affiliated psychiatric departments in France (Mondor Hospital, Créteil, University Paris-Est, and CHU Clermont-Ferrand, University of Clermont-Ferrand). All the participants were aged 18 or older at enrollment and received a diagnosis of schizophrenia by two independent psychiatrists, confirmed after an interview with the Diagnostic Interview for Genetic Studies.^12^ Diagnoses were established using the Structured Clinical Interview for DSM-IV (SCID; First^13^). Inclusion criteria were (i) diagnosis of schizophrenia according to the DSM-IV (ii) Caucasian origin (iii) antipsychotic monotherapy initiation with olanzapine or risperidone (iv) a Positive and Negative Symptoms Scale (PANSS) score^14^ >70 and a Brief Psychiatric Rating Scale (BPRS) score >45 at baseline.^15^ Exclusion criteria were (i) contraindication to a treatment by olanzapine or risperidone, (ii) a current severe somatic condition at inclusion and (iii) a history of resistant schizophrenia. All the participants were included at their first day of hospitalization and were all free of antipsychotic medication at least for 4 weeks before inclusion. After inclusion, patients received a monotherapy of antipsychotic drug either by olanzapine or risperidone. Treatment was chosen according to the discretion of the clinician.

### Outcome variables: phenotype analysis

Clinical response was prospectively assessed after 6 weeks of treatment (day 42) by assessing change in the overall score of the PANSS and the BPRS. This period is generally considered to be the most appropriate to observe the initial clinical response to treatment. The 30-item PANSS rating scale is a very well-documented scoring tool, assessing a wide range of psychopathology in SZ.^14^ In this study, it was carried out by clinically trained psychiatrists at the conclusion of a chart review and a semi-structured interview.^14^ The data were further refined by taking into account four of the analytically derived PANSS factor component scores into total, general psychopathology, positive and negative scores.

### SNP array-based genotyping

DNA from 104 patients was analyzed by a custom-made SNP array (Illumina, San Diego, CA, USA), herein designated ‘immune-inflammatory-pharmacogenetic SNP array'. This array consisted of 16 561 SNPs derived from 1998 polymorphic genes (Supplementary Table 1) selected for their implications in drug transport/metabolism/target and brain receptors as well as those involved in immune-inflammatory pathways (HLA, chemokines, cytokines, signal transduction, apoptosis).

### Quality control

The crude genotype data were analyzed using the Genome Studio software (version 1.6.3, Illumina). After quality control, removal of SNPs with missing data superior to 5%, minor allele frequency <1%, and departing from the Hardy–Weinberg equilibrium (*P*<1x10^-6^), led to a total of 14 280 remaining SNPs.

### Analysis of population stratification

To bring corrections for possible population stratification, genotypes were analyzed using the EIGENSTRAT utility of the EIGENSOFT package version 4.2 (ref. 16), which pointed out 15 outliers removed for the analysis. The top two Eigen vectors obtained from the 89 remaining patients were used as covariates to correct for population substructure in the association analysis.^16^

### Statistical analysis

For the 14 280 SNPs and 89 patients remaining after quality controls, association between genotypes and drug response was tested. The statistical analysis was performed by a multivariate linear regression using the PLINK software^17^ in the additive, dominant and recessive genotype models for the difference of various scales measuring psychotic status between day 0 (start of treatment) and day 42: delta BPRS score, delta PANSS total score, as well as the delta for the positive, negative and general subscores of the PANSS. The medication (olanzapine or risperidone) was also used as a covariate. The *P*-values were adjusted by the Bonferroni correction (statistical threshold=3.5 × 10^−6^).

### Linkage disequilibrium

For each SNP exhibiting a significant association, we examined its linkage disequilibrium (LD) profile (*r*^2^>0.8) in the 1000 genomes Phase I integrated variant set^18^ for the population of western European ancestry to identify the genes involved in the associations. Assignment of a given SNP to a gene was based on its location within the gene or within a 2-kb flanking region (encompassing potential regulatory sequences). Otherwise, the SNP was considered intergenic. The LD profile of the 1000 genomes population of western European ancestry and the herein studied patient population was quite comparable. We computed the LD with the PLINK software.^17^

### Imputation of *HLA* variants

We used the software SNP2HLA^19^ to impute the *HLA* class I and class II gene alleles and the predicted amino-acid changes. Both SNPs and indels of the region were imputed. This imputation was made possible because the *HLA* region was very well covered by the customized chip SNPs. The 6 764 imputed variants in the *HLA* region were then tested for association with drug response by a multivariate linear regression analysis (PLINK software) in the additive, dominant and recessive models, taking the two first Eigenstrat principal components as covariates. We added these additional variants to define a new Bonferroni threshold (2.37 × 10^−6^). The quality of the imputation of the *HLA* alleles was experimentally confirmed with consistent results at four digits (data not shown) by Luminex HD.

### Bioinformatics *in silico* exploration

To further explore the signals from GWAS, we performed *in silico* searches for possible SNP alterations, using several databases. Gene expression: Genevar (http://www.sanger.ac.uk/resources/software/genevar/), mRNA by SNP Browser (http://www.sph.umich.edu/csg/liang/asthma/) and GHS Express (http://genecanvas.ecgene.net/uploads/ForReview/); methylation: Genevar (http://www.sanger.ac.uk/resources/software/genevar/); polyadenylation regions: PolyApred (http://www.imtech.res.in/raghava/polyapred/submission.html); transcription factor binding sites: regulomedb (http://regulomedb.org/ derived from TRANSFAC database); miRNA genes or miRNA targets: miRBAse (http://www.mirbase.org/), miRTarBase (http://mirtarbase.mbc.nctu.edu.tw) and MicroCosm Targets (http://www.ebi.ac.uk/enright-srv/microcosm/htdocs/targets/v5/); ENCODE data: regulomedb (http://regulomedb.org/).

## Results

**Figure 1:**
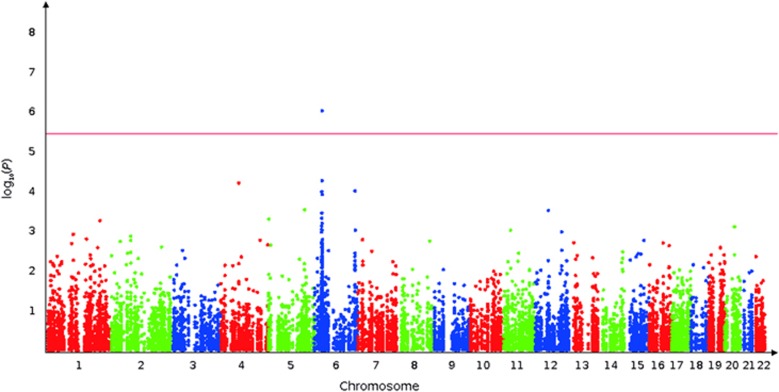
Manhattan plot depicting the distribution of the −log_10_(*P*) along the chromosomes. Manhattan plot depicting the distribution of the −log_10_(*P*) values for all the SNPs along the chromosomes measuring their association with clinical response to treatment in schizophrenia, according to the delta PANSS general and in the dominant model. PANSS, Positive and Negative Symptoms Scale; SNP, single-nucleotide polymorphism.

**Figure 2:**
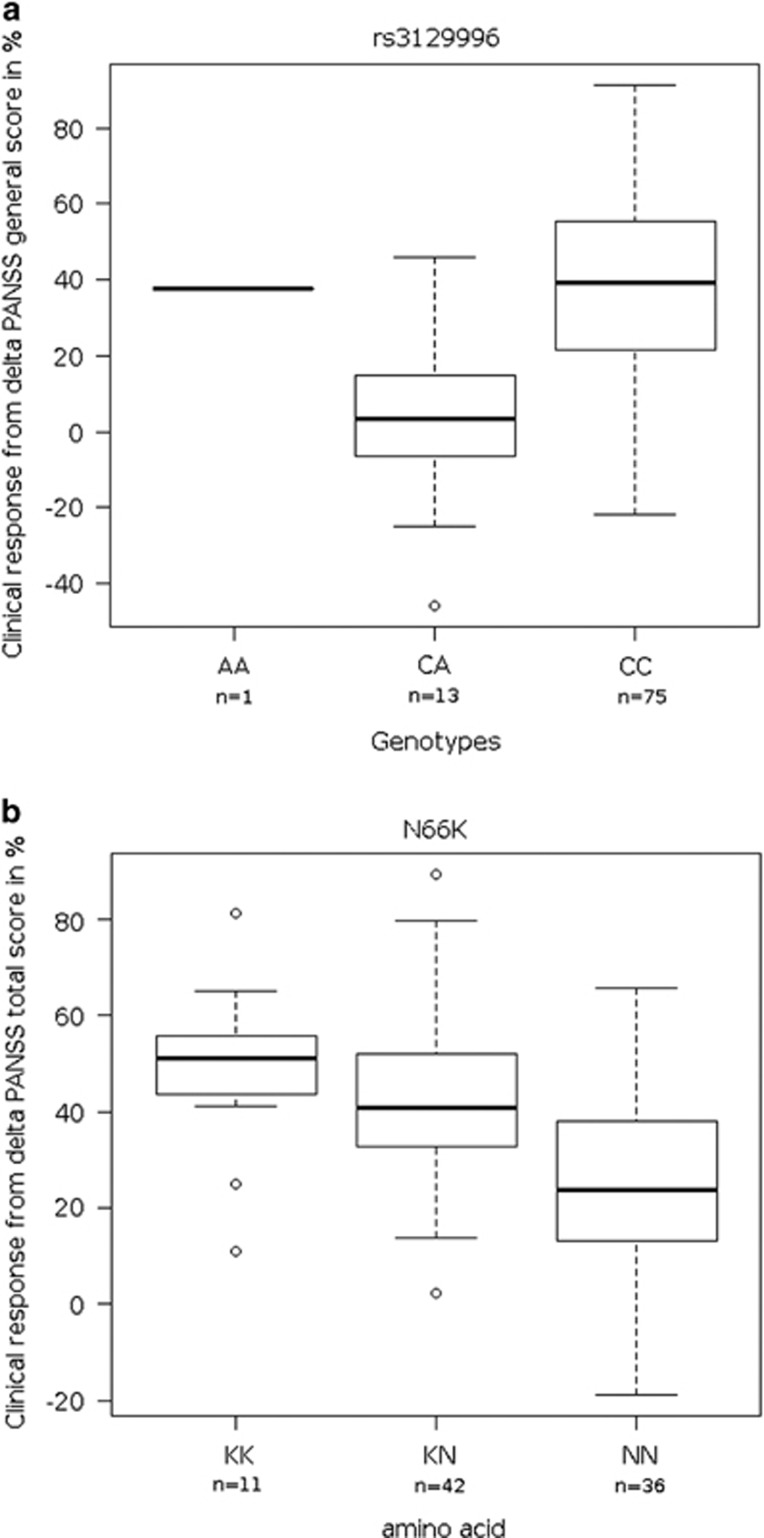
Association boxplot. (**a**) Association of rs3129996 SNP: boxplot representing clinical response to treatment in schizophrenia according to the delta PANSS general in function of the rs3129996 SNP genotypes. (**b**) Association of residue 66 in HLA-A protein: boxplot representing clinical response to treatment in schizophrenia according to the delta PANSS total in function of the amino acid at the position 66 (K or N) of the HLA-A protein (we only show the boxplot for N66K, because G62/E62 and K66 variations are totally correlated). HLA, human leukocyte antigen; PANSS, Positive and Negative Symptoms Scale; SNP, single-nucleotide polymorphism.

Overall, 14 280 SNPs passed the quality-control criteria for 89 SZ patients (66 males and 23 females) and were included in the subsequent genetic association studies. The demographics and clinical characteristics of the patients included in the analysis are described in Supplementary Table 2. We found that one SNP, namely the rs3129996, with a *P*-value of 4.68x10^−7^ passed the Bonferroni threshold (3.5 × 10^−6^) for the delta PANSS general score in the dominant model as shown in the Manhattan plot that depicts the distribution of the −log_10_(*P*) along the chromosomes (Figure 1). Moreover, This SNP (rs3129996) also exhibited weak *P*-values for other scores: *P*=4.03 × 10^−6^ for delta PANSS total score, *P*=1.76 × 10^−5^ for delta PANSS positive score, *P*=1.34 × 10^−5^ for delta BPRS score, with the exception of delta PANSS negative score (*P*=3.67 × 10^−1^; Table 1). This SNP is located in the intronic region of the *PPP1R18* gene on chromosome 6, not far from the *HLA* locus (~500 kb downstream from *HLA-C*). The frequency of the rs3129996-A allele was 8.53% in our patient population, comparable to that observed in the Caucasian panel of 1000 Genomes project^18^ (11.2%) and was associated with a lesser response to drug treatment (Figure 2a). According to the 1000 genomes project, the rs3129996 SNP is in high LD (*r*^2^⩾0.8) with seven other SNPs (rs3131110, rs3132611, rs3130245, rs3130000, rs3094092, rs3132587 and rs3094124) located in five genes (*ABCF1*, *DHX16*, *NRM*, *MDC1* and *IER3*, respectively) and in intergenic regions (Table 2). It is important to note that the nature of the treatment (olanzapine or risperidone) did not impact the association of rs3129996 as shown by the linear regression using treatment as a covariate.

The Manhattan plot of Supplementary Figure 1 zooms on the imputed SNPs and indels within 500 kb of SNP rs3129996 (see Materials and Methods). As expected, the eight aforementioned SNPs in high LD exhibited lowest *P*-values and passed, or almost passed the Bonferroni threshold for the delta PANSS general score. Interestingly, this SNP block seems to influence the expression of several genes according to two eQTL databases namely ‘GHS Express' and ‘Genevar Muther' (see Materials and Methods). The rs3130000-A allele (corresponding to rs3129996-A) is associated with a highly significant reduction in the expression of both *ABCF1* gene (*P*-values *P*=8.06 × 10^−11^ and *P<*10^−8^, in the respective databases) and *HCG27* gene (*P*-values *P*=2.17 × 10^−17^ and *P*<10^−5^, respectively). According to the Genevar database, this SNP block would also impact significantly the methylation of the CpG island (chr6:30654392-30654934) of the *PPP1R18* gene (*P*<10^−3^).

**Figure 3:**
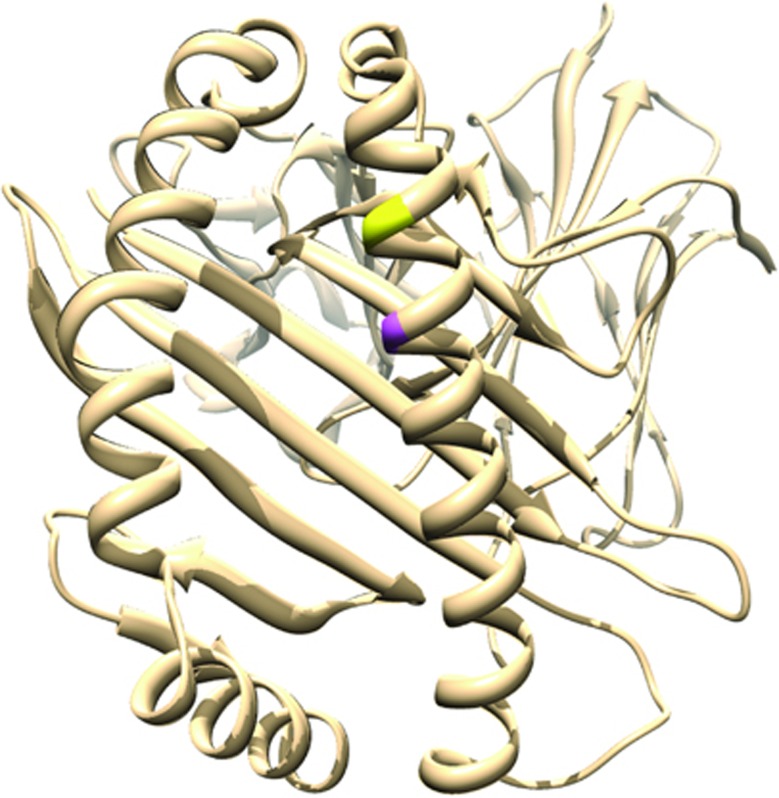
HLA-A*02 protein 3D structure. HLA-A*02 protein 3D structure (PDB ID: 4UQ3) with the amino-acid residue at position 62 highlighted in yellow and the amino-acid residue at position 66 highlighted in magenta. It is important to note that other HLA-A carry these variations and the structures could be slightly different. HLA, human leukocyte antigen.

Using the SNP2HLA software,^19^ we imputed the *HLA* class I and class II gene alleles and the corresponding amino-acid variations. The quality of the imputation of the *HLA* alleles was experimentally confirmed with consistent results at four digits (data not shown). Two amino-acid variants at positions 62 and 66 of the HLA-A protein (heavy chain), in total LD with each other and with a frequency of 35.9%, passed the Bonferroni threshold for the delta PANSS total score in the dominant model (*P*=4.87 × 10^−7^). This double variant (with either Gly or Glu residues at positions 62 (G62/E62), and Lys at position 66 (K66)) is associated with an improved response to antipsychotic drugs (Figure 2b). It also exhibited low *P*-values for other deltas, the only exception being for the delta PANSS negative score (Table 1). Amino acids at positions 62 and 66 lie within the HLA-A peptide-binding groove of the alpha 1 domain (Figure 3). These amino-acid variants are carried by several HLA-A classic alleles, the most frequent being HLA-A*02 (*D*′=1 and *r*^2^=0.6 on the whole cohort). Indeed, the association of HLA-A*02 with drug response was the strongest (*P*=5.59x10^-5^) among all *HLA* class I and II alleles. The frequencies of the two polymorphisms associated in this study are quite different (rs3129996-A ~8% G62/E62 or K66 ~35%) and there is a weak LD between the major allele of rs3129996-C and the amino-acid variants (*r*^2^=0.03 and *D*′=0.734): thus the associations observed for rs3129996 and for the amino acids go in opposite directions for their minor allele (Figure 2).

## Discussion

We have performed a customized array-based candidate gene association study of antipsychotic treatment response to SZ on a prospective cohort of patients treated with a single antipsychotic drug (olanzapine or risperidone). We proceeded with two types of analyses: (1) a search for associations with the individual SNPs of the customized chip and (2) a search for associations on variants imputed from *HLA* class I and II gene loci.

In the individual SNP analysis, we found an association between the SNP rs3129996 (6p21) and response to antipsychotic drugs, as measured by the difference between day 0 (start of treatment) and day 42 of the PANSS general score that evaluates the psychotic status. The allele rs3129996-A was associated with a lower response to drug. This SNP is located in the *PPP1R18* locus and is in LD with numerous SNPs in surrounding genes: *ABCF1, DHX16*, *NRM*, *MDC1*, *IER3*. Interestingly, the allele rs3129996-A is associated with a reduced expression of *ABCF1* according to two eQTL databases. A transcriptomic study has also associated a reduced expression of *ABCF1* with increased susceptibility to SZ.^20^ The protein encoded by *ABCF1* belongs to a family of ATP-binding cassette (ABC) transporters of molecules across different cell compartments, and has previously been associated with autoimmune disorders and inflammation.^21^ The *PPP1R18* gene encodes a phostensin, named protein Phosphatase 1 regulatory subunit 18. The expression of *PPP1R18* in the brain and its potential role in synaptic plasticity via interaction with the phosphatase PP1 (ref. 22) could also point out *PPP1R18* as a relevant target gene in Schizophrenia. The previous hypotheses must be taken with caution until their experimental confirmation. The other genes potentially involved in the SNP association by LD, namely *HCG27*, *DHX16*, *NRM*, *IER3*, exhibit no obvious explanation.

Two GWAS have previously been published on response to drug treatment in SZ.^7, 8^ One study reported an association in an intergenic region of the 4p15 locus and the other study identified an association with SNPs located in the *PDE7B* gene (6q23.3). Owing to the extreme variability of the *HLA* locus across populations, it is not a surprise that their results differ from ours. Several GWAS on susceptibility to SZ have also been published, most of them pointing out SNP associations in the *HLA* locus.^9, 10, 23^ The *HLA* SNPs involved in the main associations described by these GWAS on susceptibility to SZ exhibit a low LD with the herein identified rs3129996 SNP.

Using the software SNP2HLA, we imputed the *HLA* class I and class II variants as well as the corresponding protein amino acids. A striking association was found with the delta PANSS total score, assuming a favorable response to antipsychotic treatment in the presence of two amino-acid changes at positions 62 and 66 within the peptide-binding groove of HLA-A (Figure 3). The variant at position 66 is a nonsynonymous change, namely an Asn to a Lys. Position 62 exhibits highly variable amino acids in the population,^18^ nevertheless only a Gly or a Glu are found in presence of the K66. It is important to emphasize that this double variant encompasses almost all the carriers of the HLA-A*02 allele, which yielded the strongest *HLA* class I and II allele association found in our study. HLA-A*02, HLA-B*35 and *HLA-DQB1* have previously been associated with response to treatment in SZ.^23, 24^ The HLA-A*02 association was found in a Caucasian population of 31 individuals^25^ and its effect was consistent with our study. These converging results in two independent cohorts suggest a potent effect of HLA-A*02 and more generally of the HLA-A K66 and/or G62/E62 variants on response to treatment. The position 62 of HLA-A is highly relevant, as it plays a role in the T-cell receptor recognition according to several studies.^26^ A possible mechanism underlying this association could be that the association observed for the double amino-acid variants might correspond to a selective alleviation/modulation of the global inflammatory processes at work in SZ. It is important to note that there exists a weak LD between the major allele of rs3129996-C and the amino-acid variants (*r*^2^=0.03, *D*′=0.734) and thus the associations observed go in opposite directions for their minor allele.

Despite the relatively small size of the population (89 patients), our genetic study allowed the identification of significant associations pointing again towards to a key role of the *HLA* locus in response to treatment. The dual role found for the *HLA* locus in susceptibility to SZ (by previous genetic studies) and in treatment response (by our own study) may reflect the now well-admitted underlying dysimmune background associated with SZ, otherwise known to be alleviated by antipsychotics.^27^ There is a growing recognition that immune system dysfunction has a central role in the etiopathogenesis of schizophrenia, with strong links with autoimmune disorders.^28^ The drugs could thus act at the level of the known brain signalization pathways and/or act at the level of the inflammatory status.^29^

In conclusion, our study on antipsychotic treatment response has pointed to two novel signals in the *HLA* locus: the rs3129996 SNP in *ABCF1* and the HLA-A G62/E62 and K66 variant (notably carried by the HLA-A*02 allele). Although replication in larger trans-geographic cohorts together with functional investigations are warranted, our data are in line with the current notion that major psychotic disorders must have immune-inflammatory underpinnings, which could be targets for future personalized treatments.

## Figures and Tables

**Table 1:** *P*-values from genetic association analysis in the dominant model of clinical response to treatment in schizophrenia, for all the scores studied

*Polymorphisms*	*Delta PANSS general*	*Delta BPRS*	*Delta PANSS positive*	*Delta PANSS negative*	*Delta PANSS total*
rs3129996	4.68 × 10^−7^	1.34 × 10^−5^	1.76 × 10^−5^	3.67 × 10^−1^	4.03 × 10^−6^
AA_A_62_GE^a^	3.07 × 10^−6^	4.46 × 10^−6^	7.71 × 10^−6^	2.50 × 10^−2^	4.87 × 10^−7^
AA_A_66_K^b^	3.07 × 10^−6^	4.46 × 10^−6^	7.71 × 10^−6^	2.50 × 10^−2^	4.87 × 10^−7^

Abbreviations: BPRS, Brief Psychiatric Rating Scale; PANSS, Positive and Negative Symptoms Scale.

a AA_A_62_GE: glycine or glutamic acid at position 86 of human leukocyte antigen (HLA)-A.

b AA_A_66_K: lysine at position 90 of HLA-A.

**Table 2:** Location of SNPs in high linkage disequilibrium with rs3129996

*SNP*	*Chr*	*Pos*	*Gene*	*Type*
rs3131110	6	30385198		Intergenic
rs3132611	6	30541852	*ABCF1*	Intron
rs3130245	6	30564343		Intergenic
rs3130000	6	30628082	*DHX16*	Intron
**rs3129996**	**6**	**30651587**	***PPP1R18***	**Intron**
rs3094092	6	30657015	*NRM*	Intron
rs3132587	6	30685066	*MDC1*	5'-UTR
rs3094124	6	30711805	*IER3*	Exon

Abbreviation: Chr, chromosome; Pos, location of the SNP on the GRCh37 version genome assembly from NCBI; SNP, single-nucleotide polymorphism; UTR, untranslated region.

The rs3129996 SNP is in bold.

## Supplementary Material

Supplementary Informations
