# Supplementary material for: A double amino-acid change in the HLA-A peptide-binding groove is associated with response to psychotropic treatment in patients with schizophrenia
Source: Transl Psychiatry. 2015 Jul 28;5(7):e608–. doi: 10.1038/tp.2015.97 (PMC5068718; doi:10.1038/tp.2015.97)

Supplementary material:
A double amino acid change in the HLA-A peptide-binding groove is associated with response to psychotropic treatment in patients with schizophrenia

Supplementary Table 1: The 1998 genes covered by the custom-made SNP array.

ABCA1	*ABCG4*	*AHR*	*ANKHD1*	*ATXN7L3*	*BMF*	*C2*
ABCA10	*ABCG5*	*AHRR*	*ANKRD11*	*AURKA*	*BMP2*	*C20orf108*
ABCA11P	*ABCG8*	*AIF1*	*ANKRD36*	*AVEN*	*BMP4*	*C20orf30*
ABCA12	*ABL1*	*AIFM1*	*ANKRD40*	*AXIN1*	*BMP5*	*C22orf16*
ABCA13	*ACAD11*	*AIM1L*	*AOF1*	*AXL*	*BMP6*	*C22orf24*
ABCA3	*ACBD4*	*AIRE*	*AOX1*	*B3GALT4*	*BMP7*	*C22orf37*
ABCA5	*ACCN3*	*AKAP5*	*AOX2*	*B3GNT4*	*BMP8B*	*C2orf61*
ABCA6	*ACE*	*AKAP9*	*AP2M1*	*B3GNT6*	*BMPR1A*	*C2orf63*
ABCA7	*ACE2*	*AKR1A1*	*AP3B1*	*BAD*	*BMPR2*	*C3*
ABCA8	*ACHE*	*AKR1B1*	*AP3M2*	*BAG5*	*BRAF*	*C3orf15*
ABCA9	*ACIN1*	*AKR1B10*	*AP3S1*	*BAK1*	*BRCA1*	*C3orf22*
ABCB1	*ACSL6*	*AKR1C1*	*APAF1*	*BARD1*	*BRCA2*	*C3orf54*
ABCB11	*ACVR1*	*AKR1C2*	*APC*	*BAT1*	*BRD2*	*C4A*
ABCB4	*ADAD1*	*AKR1C3*	*APEX1*	*BAT2*	*BRPF1*	*C4B*
ABCB5	*ADAM17*	*AKR1C4*	*API5*	*BAT3*	*BSN*	*C6*
ABCB6	*ADAMTS13*	*AKT1*	*APOA4*	*BAT4*	*BTK*	*C6orf10*
ABCB7	*ADD1*	*AKT2*	*APOA5*	*BAT5*	*BTNL2*	*C6orf100*
ABCB8	*ADH1A*	*ALAD*	*APOB*	*BAX*	*BTRC*	*C6orf125*
ABCB9	*ADH1B*	*ALDH16A1*	*APOC2*	*BBC3*	*BYSL*	*C6orf134*
ABCC1	*ADH1C*	*ALDH18A1*	*APOE*	*BBS7*	*C10orf2*	*C6orf136*
ABCC10	*ADH4*	*ALDH1A1*	*APOM*	*BCHE*	*C11orf10*	*C6orf15*
ABCC11	*ADH5*	*ALDH1A2*	*AR*	*BCL2*	*C11orf74*	*C6orf205*
ABCC12	*ADH6*	*ALDH1A3*	*ARD1A*	*BCL2A1*	*C11orf9*	*C6orf21*
ABCC13	*ADH7*	*ALDH1B1*	*ARFRP1*	*BCL2L1*	*C12orf60*	*C6orf26*
ABCC2	*ADHFE1*	*ALDH1L1*	*ARHGEF6*	*BCL2L10*	*C14orf119*	*C6orf27*
ABCC3	*ADRA1B*	*ALDH1L2*	*ARL6IP5*	*BCL2L11*	*C14orf153*	*C6orf47*
ABCC4	*ADRA1D*	*ALDH2*	*ARSA*	*BCL2L12*	*C14orf172*	*C6orf48*
ABCC5	*ADRA2A*	*ALDH3A1*	*ART4*	*BCL2L13*	*C15orf33*	*C6orf66*
ABCC6	*ADRA2B*	*ALDH3A2*	*ARVCF*	*BCL2L2*	*C16orf35*	*C7*
ABCC8	*ADRA2C*	*ALDH3B1*	*ASAHL*	*BCL3*	*C16orf53*	*C8A*
ABCC9	*ADRB1*	*ALDH3B2*	*ASNA1*	*BCLAF1*	*C16orf57*	*C8B*
ABCD1	*ADRB2*	*ALDH4A1*	*ATF2*	*BCR*	*C17orf46*	*C9*
ABCD2	*AEBP1*	*ALDH5A1*	*ATF4*	*BDKRB1*	*C17orf65*	*C9orf29*
ABCD4	*AGPAT1*	*ALDH6A1*	*ATG12*	*BDKRB2*	*C17orf72*	*C9orf43*
ABCE1	*AGPAT2*	*ALDH7A1*	*ATG7*	*BDNF*	*C19orf25*	*C9orf7*
ABCF1	*AGPS*	*ALDH8A1*	*ATG9A*	*BDNFOS*	*C19orf29*	*CABP5*
ABCF2	*AGTR1*	*ALKBH2*	*ATG9B*	*BID*	*C19orf44*	*CACNA1F*
ABCF3	*AGTR2*	*ALPI*	*ATM*	*BIRC4*	*C19orf48*	*CACNA1G*
ABCG1	*AGXT*	*ALS2CR12*	*ATP6V1G2*	*BIRC5*	*C1orf167*	*CACNG7*
ABCG2	*AHNAK*	*ANAPC10*	*ATP7B*	*BIVM*	*C1QTNF5*	*CALCP*

CALM1	*CCND3*	*CDKN2D*	*CNN2*	*CYP1A2*	*D6S2723E*	*EFNB3*
CALM2	*CCNE1*	*CDSN*	*CNTD1*	*CYP1B1*	*DAB2*	*EGF*
CALML4	*CCNE2*	*CECR6*	*COASY*	*CYP21A2*	*DAO*	*EGFL7*
CALR	*CCNG1*	*CECR7*	*COL11A2*	*CYP24A1*	*DAOA*	*EGFR*
CALR3	*CCNH*	*CERKL*	*COL18A1*	*CYP26A1*	*DAXX*	*EGLN2*
CAMK1	*CCNO*	*CES1*	*COL1A1*	*CYP26B1*	*DCK*	*EGLN3*
CAMK2A	*CCR3*	*CES2*	*COL20A1*	*CYP26C1*	*DCN*	*EHMT2*
CAMKK1	*CCR4*	*CES7*	*COMT*	*CYP27A1*	*DCTD*	*EIF2A*
CANX	*CCR6*	*CETP*	*CORO1C*	*CYP27B1*	*DDAH2*	*EIF4A3*
CAPN5	*CCR7*	*CFB*	*CPB2*	*CYP2A13*	*DDOST*	*EIF4E1B*
CAPZA3	*CCR8*	*CFI*	*CREB1*	*CYP2A6*	*DDR1*	*ELK1*
CARD10	*CCR9*	*CFLAR*	*CREB3*	*CYP2A7*	*DDX39*	*ELK3*
CARD14	*CD14*	*CFTR*	*CREBBP*	*CYP2B6*	*DEPDC4*	*ELMO3*
CARD6	*CD27*	*CG018*	*CREBL1*	*CYP2B7P1*	*DEXI*	*ELP2*
CARD8	*CD274*	*CHEK1*	*CRELD1*	*CYP2C18*	*DGCR6*	*ENOSF1*
CARD9	*CD28*	*CHEK2*	*CRIP3*	*CYP2C19*	*DHFR*	*ENTHD1*
CASP1	*CD3D*	*CHKA*	*CRIPT*	*CYP2C8*	*DHX29*	*EP300*
CASP10	*CD3E*	*CHMP5*	*CRK*	*CYP2C9*	*DIABLO*	*EP400*
CASP14	*CD3EAP*	*CHRD*	*CRKL*	*CYP2D6*	*DIAPH1*	*EPAS1*
CASP2	*CD3G*	*CHRM1*	*CROT*	*CYP2E1*	*DKK4*	*EPHX2*
CASP3	*CD4*	*CHRM2*	*CSF1R*	*CYP2F1*	*DNAH1*	*EPM2AIP1*
CASP4	*CD40*	*CHRNA2*	*CSF2*	*CYP2F1P*	*DNAJC4*	*EPS15L1*
CASP5	*CD40LG*	*CHRNA3*	*CSF3*	*CYP2G1P*	*DNMT1*	*ERBB2*
CASP6	*CD52*	*CHRNA4*	*CSF3R*	*CYP2G2P*	*DNMT3A*	*ERBB3*
CASP7	*CD59*	*CHRNA5*	*CSGlcA-T*	*CYP2J2*	*DNMT3B*	*ERBB4*
CASP8	*CD69*	*CHRNA6*	*CSMD1*	*CYP2R1*	*DNMT3L*	*ERCC1*
CASP9	*CD70*	*CHRNA7*	*CSN1S1*	*CYP2S1*	*DNTTIP1*	*ERCC2*
CAT	*CD80*	*CHRNB4*	*CSRP2P*	*CYP2T2P*	*DOK2*	*ERCC3*
CBL	*CD86*	*CHST1*	*CSTF1*	*CYP2T3P*	*DOK4*	*ERCC4*
CBR1	*CD8A*	*CHST10*	*CTGF*	*CYP2U1*	*DOM3Z*	*ERCC5*
CBR3	*CD8B*	*CHST11*	*CTLA4*	*CYP2W1*	*DPCR1*	*ERCC6*
CBX4	*CD97*	*CHST12*	*CTNNA1*	*CYP39A1*	*DRD1*	*ERCC8*
CCBP2	*CDA*	*CHST13*	*CTNNB1*	*CYP3A4*	*DRD2*	*ESM1*
CCDC101	*CDC2*	*CHST2*	*CTSA*	*CYP3A43*	*DRD3*	*ESPN*
CCDC109B	*CDC20*	*CHST3*	*CTSD*	*CYP3A5*	*DTNBP1*	*ESR1*
CCDC111	*CDC25A*	*CHST4*	*CTSG*	*CYP3A7*	*E2F1*	*ESR2*
CCDC22	*CDC25C*	*CHST5*	*CTSL1*	*CYP46A1*	*E2F2*	*EVPL*
CCDC33	*CDH1*	*CHST6*	*CUL1*	*CYP4A11*	*E2F3*	*EXOC3L*
CCDC65	*CDH15*	*CHST7*	*CX3CR1*	*CYP4A22*	*E2F4*	*EZH2*
CCDC88B	*CDH5*	*CHST8*	*CXADR*	*CYP4B1*	*E2F5*	*F10*
CCDC97	*CDK2*	*CHST9*	*CXCL12*	*CYP4F11*	*E2F6*	*F11*
CCHCR1	*CDK3*	*CIITA*	*CXCL9*	*CYP4F12*	*E2F7*	*F12*
CCL1	*CDK4*	*CKLF*	*CXCR4*	*CYP4F2*	*E2F8*	*F2*
CCL13	*CDK6*	*CKS1BP*	*CYB5R3*	*CYP4F22*	*EDN1*	*F2R*
CCL2	*CDK7*	*CLCN6*	*CYBA*	*CYP4F3*	*EDNRA*	*F7*
CCL27	*CDK8*	*CLEC2D*	*CYLD*	*CYP4F8*	*EDNRB*	*F8*
CCL3	*CDKN1A*	*CLIC1*	*CYP11A1*	*CYP4X1*	*EEF1A1*	*F9*
CCL4	*CDKN1B*	*CLPTM1*	*CYP11B1*	*CYP4Z1*	*EEF1A2*	*FAM101A*
CCL5	*CDKN1C*	*CLTA*	*CYP11B2*	*CYP51A1*	*EEF1D*	*FAM46A*
CCNB1	*CDKN2A*	*CLTB*	*CYP17A1*	*CYP7A1*	*EFNA2*	*FAM54A*
CCNB2	*CDKN2B*	*CLTCL1*	*CYP19A1*	*CYP7B1*	*EFNB1*	*FAM55C*
CCND1	*CDKN2C*	*CNDP1*	*CYP1A1*	*CYP8B1*	*EFNB2*	*FAM82A*

FAM83E	*FOLR1*	*GLDN*	*HASNT*	*HLA-DRA*	*ICOSLG*	*IL4R*
FAM83H	*FOLR2*	*GLI1*	*HBEGF*	*HLA-DRB1*	*ID3*	*IL5*
FAS	*FOS*	*GLRA1*	*HCG22*	*HLA-DRB5*	*IFITM4P*	*IL6*
FBF1	*FOSB*	*GLS*	*HCG27*	*HLA-DRB9*	*IFNA1*	*IL7*
FBXO11	*FOSL1*	*GML*	*HCG2P6*	*HLA-E*	*IFNB1*	*IL7R*
FBXO16	*FOXP3*	*GMNN*	*HCG4*	*HLA-F*	*IFNG*	*IL8RA*
FBXO40	*FRAP1*	*GNB3*	*HCG4P10*	*HLA-G*	*IFNGR1*	*IL9*
FBXW7	*FRK*	*GNB5*	*HCG4P11*	*HLA-H*	*IFT122*	*ILK*
FCER2	*FRS3*	*GNE*	*HCG4P3*	*HLA-L*	*IGF1*	*IMPDH1*
FCGRT	*FURIN*	*GNL1*	*HCG4P4*	*HLA-U*	*IGF1R*	*IMPDH2*
FCHSD1	*FUT7*	*GOLGB1*	*HCG4P5*	*HMGCL*	*IGF2*	*INE1*
FEN1	*FVT1*	*GORASP1*	*HCG4P9*	*HMGCR*	*IGF2AS*	*INHBE*
FGA	*FYCO1*	*GP5*	*HCG9*	*HMOX1*	*IGF2R*	*INS-IGF2*
FGB	*FZD3*	*GPC2*	*HCG9P5*	*HNF1A*	*IGFBP1*	*INSL3*
FGF1	*G6PC3*	*GPLD1*	*HCK*	*HNMT*	*IGFBP2*	*INSR*
FGF2	*G6PD*	*GPR126*	*HCLS1*	*HOM-TES-103*	*IGFBP3*	*INTS8*
FGF5	*GAB1*	*GPR162*	*HCP5*	*HPRT1*	*IGFBP4*	*IRF4*
FGF6	*GAB2*	*GPR63*	*HCP5P10*	*HRH1*	*IGFBP5*	*IRF5*
FGF7	*GAB3*	*GPSM1*	*HCP5P13*	*HRH2*	*IGFBPL1*	*IRS1*
FGF9	*GAB4*	*GPSM3*	*HCP5P14*	*HRH4*	*IGHEP2*	*ITGA2*
FGFBP2	*GABBR1*	*GPX1*	*HCP5P2*	*HS3ST1*	*IGSF6*	*ITGA2B*
FGFR2	*GABRA1*	*GPX2*	*HCP5P3*	*HSD17B1*	*IHPK3*	*ITGA3*
FGFR3	*GABRA2*	*GRAP*	*HCP5P6*	*HSD17B13*	*IKBKB*	*ITGA4*
FGG	*GABRA3*	*GRAP2*	*HDAC10*	*HSD17B2*	*IL10RA*	*ITGA5*
FIGF	*GABRA4*	*GRB2*	*HDAC11*	*HSD17B3*	*IL11RA*	*ITGA6*
FKBP1A	*GABRA5*	*GRHPR*	*HDAC2*	*HSD17B4*	*IL12A*	*ITGAD*
FKBP1B	*GABRA6*	*GRIK2*	*HDAC3*	*HSD17B6*	*IL12RB1*	*ITGAM*
FKBP2	*GABRB1*	*GRIK4*	*HDAC4*	*HSD17B8*	*IL13*	*ITGAV*
FKBP4	*GABRB2*	*GRIK5*	*HDAC5*	*HSDL1*	*IL13RA2*	*ITGAX*
FKBP5	*GABRB3*	*GRK4*	*HDAC6*	*HSP90B1*	*IL15*	*ITGB2*
FKBP6	*GABRR2*	*GSDM1*	*HDAC7A*	*HSPA1A*	*IL15RA*	*ITPR3*
FKBP8	*GAD1*	*GSK3B*	*HDAC8*	*HSPA1B*	*IL16*	*JAK1*
FKBP9	*GAD2*	*GSTA1*	*HDAC9*	*HSPC111*	*IL17A*	*JAK2*
FKBPL	*GADD45B*	*GSTA2*	*HEYL*	*HSPD1*	*IL17C*	*JAK3*
FLJ12595	*GAL3ST1*	*GSTA3*	*HGF*	*HSPG2*	*IL17RA*	*JARID1D*
FLJ20489	*GAL3ST2*	*GSTA4*	*HIF1A*	*HTATIP*	*IL18*	*JARID2*
FLJ22167	*GAL3ST3*	*GSTA5*	*HIF1AN*	*HTR1A*	*IL18R1*	*JUN*
FLJ31306	*GALT*	*GSTO1*	*HIF3A*	*HTR1B*	*IL18RAP*	*JUND*
FLJ34503	*GAPDH*	*GSTP1*	*HLA-A*	*HTR2A*	*IL1A*	*KALRN*
FLJ35024	*GAS6*	*GSTT1*	*HLA-B*	*HTR2B*	*IL1B*	*KCNE1*
FLJ35429	*GATA3*	*GSTZ1*	*HLA-C*	*HTR2C*	*IL1F10*	*KCNE2*
FLJ41484	*GBA2*	*GTF2F2L*	*HLA-DMA*	*HTR3A*	*IL1R1*	*KCNH2*
FLJ42258	*GCDH*	*GTF2H3*	*HLA-DMB*	*HTR3B*	*IL1R2*	*KCNJ11*
FLJ43752	*GFM1*	*GTF2H4*	*HLA-DOA*	*HTR4*	*IL1RL1*	*KCNK7*
FLJ45422	*GFPT1*	*GTF2I*	*HLA-DOB*	*HTR5A*	*IL1RN*	*KDR*
FLJ45983	*GGCX*	*GUSB*	*HLA-DPA1*	*HTR7*	*IL2*	*KHDRBS3*
FLOT1	*GGH*	*GUSBL1*	*HLA-DPB1*	*IAPP*	*IL22RA1*	*KIF24*
FLT1	*GGNBP1*	*GZMB*	*HLA-DPB2*	*ICAM1*	*IL23A*	*KIFC1*
FLT3	*GH1*	*H3F3B*	*HLA-DQA1*	*ICAM3*	*IL2RA*	*KIR2DL1*
FLT3LG	*GIPC3*	*HACE1*	*HLA-DQA2*	*ICAM4*	*IL3*	*KIR2DL3*
FLT4	*GJA1*	*HADH*	*HLA-DQB1*	*ICAM5*	*IL32*	*KIR2DL4*
FOLH1	*GJB2*	*HAS2*	*HLA-DQB2*	*ICOS*	*IL4*	*KIR2DP1*

KIR2DS4	*LTB4R2*	*METTL9*	*MSH6*	*NOD1*	*OR2H4P*	*PECAM1*
KIR3DL1	*LY6G5C*	*MFNG*	*MTHFD1*	*NOD2*	*OR2H5P*	*PEG10*
KIR3DL3	*LY6G6C*	*MFRP*	*MTHFD2*	*NOS1*	*OR2I1P*	*PEG3*
KIT	*LY6G6D*	*MFSD5*	*MTHFD2L*	*NOS2A*	*OR2J1*	*PF4*
KITLG	*LY6G6E*	*MGAT2*	*MTHFR*	*NOS3*	*OR2J2*	*PFKL*
KLC1	*LYPLA2P1*	*MGC45922*	*MTHFS*	*NOT*	*OR2J3*	*PGR*
KLC3	*LYVE1*	*MICB*	*MTIF2*	*NOTCH1*	*OR2J4P*	*PHF1*
KLF1	*MACROD1*	*MICD*	*MTRR*	*NOTCH3*	*OR2N1P*	*PHPT1*
KLK1	*MADD*	*MICG*	*MUTYH*	*NOTCH4*	*OR2P1P*	*PIAS1*
KLK15	*MANBA*	*MIER3*	*MVD*	*NOV*	*OR2U2P*	*PIGF*
KLRC1	*MAOB*	*MIP*	*MVP*	*NOX5*	*OR2W1*	*PIH1D1*
KLRC4	*MAP2*	*MITF*	*MYADM*	*NPHP1*	*OR5U1*	*PIK3C2A*
KLRK1	*MAP2K1*	*MKRN2*	*MYBPC3*	*NPHS1*	*OR5V1*	*PIK3CA*
KRAS	*MAP2K2*	*MLH1*	*MYC*	*NQO1*	*OR7M1P*	*PIK3CB*
KRI1	*MAP2K3*	*MLN*	*MYL8P*	*NR1H2*	*OSBPL1A*	*PIK3R1*
KRT18P1	*MAP2K4*	*MME*	*MYO7A*	*NR1H3*	*OSGEP*	*PIK3R5*
KRT8P8	*MAP2K5*	*MMP1*	*MYOM3*	*NR1H4*	*OTUB1*	*PINK1*
LAIR1	*MAP2K7*	*MMP10*	*NAB2*	*NR1I2*	*OTUB2*	*PIP*
LAMP1	*MAP3K1*	*MMP11*	*NAPRT1*	*NR2C2*	*P2RX1*	*PIP4K2B*
LAMP2	*MAP3K11*	*MMP12*	*NAT1*	*NR2C2AP*	*P2RX7*	*PIP5K1B*
LAP3	*MAP3K14*	*MMP13*	*NAT2*	*NR3C1*	*P2RY1*	*PIR*
LAT	*MAP3K2*	*MMP14*	*NBN*	*NR4A1*	*P2RY5*	*PKD2L2*
LCK	*MAP3K3*	*MMP15*	*NCAPD2*	*NRG1*	*P5.8*	*PLA2G12A*
LDHA	*MAP3K4*	*MMP17*	*NCAPH2*	*NRP1*	*PAK1*	*PLA2G4B*
LDHAL6A	*MAP3K5*	*MMP19*	*NCBP1*	*NRP2*	*PALB2*	*PLA2G4C*
LDHB	*MAP3K7IP2*	*MMP2*	*NCOA1*	*NT5C1A*	*PAN3*	*PLA2G5*
LDHC	*MAP4K2*	*MMP20*	*NCOA3*	*NT5C1B*	*PARC*	*PLA2G7*
LDLR	*MAPK1*	*MMP21*	*NCOR1*	*NT5C2*	*PARK7*	*PLAT*
LDLRAD2	*MAPK11*	*MMP24*	*NCOR2*	*NT5C3*	*PBX2*	*PLAUR*
LEF1	*MAPK12*	*MMP25*	*NCR1*	*NT5E*	*PCBP2P1*	*PLCB2*
LEMD2	*MAPK13*	*MMP26*	*NCR2*	*NT5M*	*PCGF2*	*PLCB3*
LEPREL2	*MAPK14*	*MMP27*	*NCR3*	*NTN1*	*PCNA*	*PLCD1*
LIG1	*MAPK15*	*MMP28*	*NDUFB8*	*NUB1*	*PCNXL3*	*PLCD3*
LIG3	*MAPK3*	*MMP3*	*NDUFS8*	*NUP107*	*PCSK1*	*PLCD4*
LILRB2	*MAPK6*	*MMP7*	*NECAB3*	*NUP93*	*PCSK4*	*PLCG1*
LIN28	*MAPK8*	*MMP8*	*NEU1*	*OAT*	*PCSK7*	*PLCZ1*
LIN52	*MAPK8IP2*	*MMP9*	*NFKB1*	*ODF3L2*	*PCSK9*	*PLEKHG5*
LMBRD2	*MAPK9*	*MMRN2*	*NFKB2*	*OGDH*	*PCTK2*	*PLG*
LPCAT1	*MAPT*	*MOBKL1A*	*NFKBIA*	*OGG1*	*PDCD2*	*PLK1*
LPCAT2	*MARCH9*	*MOG*	*NFKBIB*	*OMP*	*PDCD2L*	*PLK2*
LPL	*MAS1L*	*MPG*	*NFKBIE*	*OR10C1*	*PDCD6*	*PLK3*
LPO	*MBD4*	*MPO*	*NFKBIL1*	*OR11A1*	*PDE3B*	*PLK4*
LRG1	*MBTPS1*	*MRE11A*	*NFKBIL2*	*OR12D1P*	*PDGFB*	*PLRG1*
LRP1	*MCAM*	*MRPL38*	*NFKBIZ*	*OR12D2*	*PDGFC*	*PLTP*
LRRC8E	*MCCD1*	*MRPL52*	*NGFR*	*OR12D3*	*PDGFRA*	*PNO1*
LRRFIP2	*MDM2*	*MRPS15*	*NKIRAS1*	*OR2AD1P*	*PDGFRB*	*PNPO*
LRRTM2	*MED12L*	*MRPS18B*	*NKIRAS2*	*OR2AE1*	*PDIA2*	*POLA2*
LSM14B	*MED24*	*MRPS25*	*NKRF*	*OR2B3P*	*PDIA4*	*POLB*
LST1	*MEN1*	*MRVI1*	*NKTR*	*OR2B4P*	*PDK1*	*POLD1*
LTA	*MERTK*	*MS4A1*	*NLRC4*	*OR2G1P*	*PDK2*	*POLD2*
LTB4DH	*MET*	*MSH2*	*NMT1*	*OR2H1*	*PDPK1*	*POLD3*
LTB4R	*METTL1*	*MSH5*	*NNMT*	*OR2H2*	*PDX1*	*POLD4*

POLDIP2	*PRR3*	*RAD51*	*RPL37*	*SERP1*	*SLC29A2*	*SOD3*
POLDIP3	*PRRT1*	*RAF1*	*RPL37P17*	*SERPINB2*	*SLC29A3*	*SOS1*
POLE	*PSD*	*RAG1*	*RPL38P1*	*SERPINB7*	*SLC29A4*	*SOS2*
POLE2	*PSMA1*	*RALBP1*	*RPL7AP7*	*SERPINE1*	*SLC2A1*	*SOSTDC1*
POLE3	*PSMA2*	*RANP1*	*RPP21*	*SERPING1*	*SLC30A6*	*SP100*
POLE4	*PSMA3*	*RAP1B*	*RPS13*	*SF3B14*	*SLC34A1*	*SP2*
POLI	*PSMA4*	*RARA*	*RPS18*	*SF3B2*	*SLC35B2*	*SPARC*
POLL	*PSMA6*	*RARG*	*RPS19BP1*	*SFTPG*	*SLC35E3*	*SPATA7*
POLR2C	*PSMA8*	*RARRES1*	*RPS24P17*	*SGCE*	*SLC38A8*	*SPESP1*
POLR2H	*PSMB1*	*RASSF1*	*RPS6KA1*	*SGMS2*	*SLC39A13*	*SPG7*
POMT2	*PSMB10*	*RAVER1*	*RPS6KA4*	*SGSH*	*SLC39A6*	*SPI1*
PON1	*PSMB2*	*RAVER2*	*RPS6KA5*	*SGTA*	*SLC39A7*	*SPNS1*
PON2	*PSMB3*	*RB1*	*RPS6KB1*	*SH3BP2*	*SLC44A4*	*SPP1*
PON3	*PSMB5*	*RBBP8*	*RPS9P1*	*SH3BP5*	*SLC4A9*	*SRC*
POR	*PSMB6*	*RBL1*	*RRM1*	*SHB*	*SLC5A6*	*SRD5A1*
POU2AF1	*PSMB7*	*RBX1*	*RRM2*	*SHC2*	*SLC6A18*	*SRD5A2*
POU5F1	*PSMB8*	*RDBP*	*RRS1*	*SHCBP1*	*SLC6A2*	*SREBF1*
PPARA	*PSMB9*	*RDH14*	*RTEL1*	*SIAH1*	*SLC6A3*	*SREBF2*
PPARD	*PSMC3*	*RELA*	*RTN2*	*SIAH2*	*SLC6A4*	*SRF*
PPARG	*PSMC4*	*RENBP*	*RXRA*	*SILV*	*SLC6A7*	*SRP14P1*
PPBP	*PSMC5*	*REXO4*	*RXRB*	*SIPA1*	*SLC7A5*	*SS18L1*
PPIA	*PSMC6*	*RFC1*	*RYR3*	*SIRT2*	*SLC7A7*	*SS18L2*
PPIAP9	*PSMD1*	*RFC2*	*SAE1*	*SKAP1*	*SLC9A3R2*	*STAM*
PPIC	*PSMD2*	*RFC3*	*SAP30BP*	*SKI*	*SLCO1A2*	*STAM2*
PPIE	*PSMD3*	*RFC4*	*SBNO1*	*SKIV2L*	*SLCO1B1*	*STAT1*
PPIH	*PSMD5*	*RFC5*	*SCARB1*	*SKP1*	*SLCO1B3*	*STAT3*
PPP1R10	*PSMD7*	*RFFL*	*SCGB1A1*	*SKP2*	*SLCO2B1*	*STAT5A*
PPP1R11	*PSMG1*	*RFXANK*	*SCN1A*	*SLC10A1*	*SLU7*	*STAT5B*
PPP1R13L	*PSORS1C1*	*RG9MTD3*	*SCN1B*	*SLC10A2*	*SMAD1*	*STAT6*
PPP1R2P1	*PSORS1C2*	*RGL2*	*SCN5A*	*SLC12A1*	*SMAD2*	*STATH*
PPP1R9A	*PTEN*	*RHEB*	*SCN8A*	*SLC12A3*	*SMAD3*	*STH*
PPP3CB	*PTGDR*	*RHOA*	*SCNN1G*	*SLC12A4*	*SMAD4*	*STIM1*
PPP3R1	*PTGES3*	*RHOB*	*SCO2*	*SLC13A1*	*SMAD6*	*STK11*
PPP5C	*PTGIS*	*RHOBTB2*	*SCYL2*	*SLC15A1*	*SMAD7*	*STK19*
PPT2	*PTGS1*	*RHOQ*	*SDCCAG3*	*SLC15A2*	*SMARCD2*	*STT3A*
PRF1	*PTH2R*	*RHOT2*	*SDHDP4*	*SLC17A6*	*SMUG1*	*SUGT1*
PRIC285	*PTHR1*	*RING1*	*SDR42E1*	*SLC17A7*	*SMURF1*	*SULF2*
PRICKLE3	*PTK2*	*RIPK1*	*SEC31B*	*SLC17A8*	*SMURF2*	*SULT1A1*
PRKACA	*PTK2B*	*RNASEH2C*	*SELPLG*	*SLC22A1*	*SNAP25*	*SULT1A2*
PRKACG	*PTMAP1*	*RNF121*	*SEMA3B*	*SLC22A2*	*SNAPC4*	*SULT1B1*
PRKAG3	*PTPN11*	*RNF175*	*SEMA3C*	*SLC22A3*	*SNCA*	*SULT1C2*
PRKCA	*PTPN21*	*RNF214*	*SEMA3D*	*SLC22A4*	*SNCAIP*	*SULT1E1*
PRKCB1	*PTTG1*	*RNF26*	*SEMA3F*	*SLC22A5*	*SNCB*	*SULT2A1*
PRKCG	*PTTG1IP*	*RNF39*	*SEMA4B*	*SLC22A6*	*SNCG*	*SULT2B1*
PRKCI	*PXN*	*RNF4*	*SEMA4D*	*SLC22A7*	*SNORA38*	*SULT4A1*
PRKCZ	*QRICH1*	*RNF5*	*SEMA4F*	*SLC22A8*	*SNORA81*	*SULT6B1*
PRKCZ	*RAB15*	*RPA1*	*SEMA4G*	*SLC25A27*	*SNORD117*	*SUMO1*
PROC	*RAB2A*	*RPL15*	*SEMA5A*	*SLC26A8*	*SNORD2*	*SUMO3*
PROCR	*RAB38*	*RPL23AP1*	*SEMA6B*	*SLC28A1*	*SNORD32B*	*SUMO4*
PRODH	*RAB4B*	*RPL23AP2*	*SEMA6D*	*SLC28A2*	*SNORD84*	*SUOX*
PROM1	*RAB5B*	*RPL31P62*	*SEMA7A*	*SLC28A3*	*SOD1*	*SYN2*
PROS1	*RAD50*	*RPL32P1*	*SERGEF*	*SLC29A1*	*SOD2*	*SYN3*

SYNGAP1	*TLR4*	*TRAF4*	*UBXD5*	*WNT10B*
SYNGR2	*TLR7*	*TRAF6*	*UCHL1*	*WNT11*
SYNPO	*TLR7-like*	*TRIM10*	*UCK1*	*WNT16*
TAGLN	*TLR9*	*TRIM15*	*UGCG*	*WNT2*
TAP1	*TMEM130*	*TRIM24*	*UGT1A1*	*WNT3*
TAP2	*TMEM139*	*TRIM26*	*UGT1A10*	*WNT4*
TAPBP	*TMEM151B*	*TRIM27*	*UGT1A2P*	*WNT5A*
TAPBPL	*TMEM167*	*TRIM31*	*UGT1A3*	*WNT5B*
TBC1D15	*TMEM55B*	*TRIM35*	*UGT1A4*	*WNT6*
TBP	*TMF1*	*TRIM39*	*UGT1A6*	*WNT7A*
TBXA2R	*TMOD3*	*TRIM40*	*UGT1A7*	*WNT7B*
TBXAS1	*TMUB1*	*TSC2*	*UGT1A8*	*WNT8A*
TCEB1P2	*TNF*	*TSC22D1*	*UGT1A9*	*WNT8B*
TCF12	*TNFAIP1*	*TSG101*	*UGT2A1*	*WNT9B*
TCF19	*TNFAIP2*	*TTC21A*	*UGT2B10*	*WSB2*
TCF7	*TNFAIP3*	*TUBB*	*UGT2B11*	*WWOX*
TCF7L1	*TNFRSF10A*	*TUBGCP6*	*UGT2B17*	*XCR1*
TCF7L2	*TNFRSF10B*	*TWF2*	*UGT2B28*	*XDH*
TCIRG1	*TNFRSF10C*	*TXNRD2*	*UGT2B4*	*XPA*
TCTN3	*TNFRSF10D*	*TYMS*	*UGT2B7*	*XPC*
TDG	*TNFRSF11B*	*TYR*	*UGT8*	*XPO7*
TERT	*TNFRSF14*	*TYRO3*	*UMPS*	*XRCC1*
TESK2	*TNFRSF17*	*TYRP1*	*UNG*	*XRCC2*
TEX12	*TNFRSF1A*	*UBA1*	*UROC1*	*XRCC3*
TF	*TNFRSF1B*	*UBA2*	*UROS*	*XRCC4*
TFDP1	*TNFRSF21*	*UBA3*	*USH1C*	*XRCC5*
TFPI	*TNFRSF25*	*UBA5*	*USP24*	*YES1*
TFRC	*TNFRSF8*	*UBA52*	*USP31*	*YWHAB*
TGFA	*TNFRSF9*	*UBA6*	*UTP6*	*YWHAE*
TGFB1	*TNFSF10*	*UBA7*	*VARS*	*YWHAG*
TGFB3	*TNFSF11*	*UBAC1*	*VARS2*	*YWHAH*
TGFBR2	*TNFSF12*	*UBAP1*	*VDR*	*YWHAQ*
TGIF1	*TNFSF12-TNFSF13*	*UBAP2*	*VEGFA*	*YWHAZ*
THBS1	*TNFSF14*	*UBASH3A*	*VEGFB*	*ZAP70*
THBS2	*TNFSF8*	*UBB*	*VEGFC*	*ZBTB12*
THBS4	*TNFSF9*	*UBC*	*VGLL4*	*ZBTB22*
THOP1	*TNPO3*	*UBD*	*VHL*	*ZBTB5*
THPO	*TNXB*	*UBE2A*	*VILL*	*ZC3H13*
THSD1	*TOP1*	*UBE2B*	*VKORC1*	*ZFAND2B*
THY1	*TOP2A*	*UBE2C*	*VLDLR*	*ZFP57*
TIMELESS	*TOP2B*	*UBE2D1*	*VPS52*	*ZHX1*
TIMP2	*TP53*	*UBE2D2*	*WDR19*	*ZHX3*
TIMP3	*TP73*	*UBE2D3*	*WDR24*	*ZIM2*
TIMP4	*TPD52*	*UBE2E1*	*WDR46*	*ZNF259*
TK1	*TPH1*	*UBE2E3*	*WDR48*	*ZNF311*
TK2	*TPH2*	*UBE2G1*	*WDR79*	*ZNF318*
TLE2	*TPMT*	*UBE2G2*	*WEE1*	*ZNF385C*
TLE4	*TPP1*	*UBE2I*	*WISP1*	*ZNF483*
TLE6	*TRADD*	*UBE2J1*	*WISP2*	*ZNF721*
TLN1	*TRAF1*	*UBL7*	*WISP3*	*ZNRD1*
TLR2	*TRAF2*	*UBR1*	*WNT1*	
TLR3	*TRAF3*	*UBR5*	*WNT10A*	

Supplementary Table 2. Demographic and clinical characteristics of the population of patients with Schizophrenia.

Variables	Whole sample N=89	
Age (in years)	33	(11.5)
Gender (male)	66	(74.2%)
Family history of schizophrenia[1]	11	(12.3%)
Lifetime personal history of depression	27	(30.3%)
Lifetime alcohol dependency	14	(15.7%)
Lifetime cannabis regular consumption	22	(24.7%)
Number of psychiatric hospitalizations[1]	3	[0-9]
Age at illness' onset (years)	23.17	(6)
Age at first psychiatric treatment (years)	25.53	(6.8)
Age at first hospitalization in psychiatry	21	(8.5)
Olanzapine treatment[2]	52	(58.4%)
Olanzapine dose (mg/day)	20.56	(11.4)
Risperidone dose (mg/day)	6.52	(3.6)
D0 PANSS total score	108.15	(25.8)
D42 PANSS total score	79.32	(23.6)
D0 PANSS positive score	27.95	(7.87)
D42 PANSS positive score	18.37	(6.9)
D0 PANSS negative score	28.59	(9.02)
D42 PANSS negative score	22.54	(7.6)
D0 PANSS general score	51.6	(13.68)
D42 PANSS general score	38.41	(12.28)
D0 BPRS score	61.47	(14.83)
D42 BPRS score	43.98	(13.79)
D0 CGI score	5·2	(0.97)
D42 CGI score	4.18	(1.26)

[1] First degree

[2] All patients were treated by olanzapine or risperidone monotherapy.

Qualitative variables are expressed by N (%) and quantitative variables by mean (standard deviation), except for discrete variables that are expressed by median (min-max). PANSS positive and negative symptoms scale. BPRS Brief psychiatric rating scale. CGI Clinical global impression.

Supplementary Figure 1: Imputation results for the *HLA* region.

At the top, Manhattan plot zooming 500 kb apart from rs3129996 (in purple) and depicting the distribution of the -$\log_{10}(P)$ for the genotyped and imputed variants (SNPs and indels). The analysis was performed in the dominant model for clinical response to treatment in schizophrenia, according to the delta PANSS general. The color of the dots represents the level of linkage disequilibrium (r^2) with rs3129996, according to the legend at the top left. At the bottom, the genes in the region are represented.

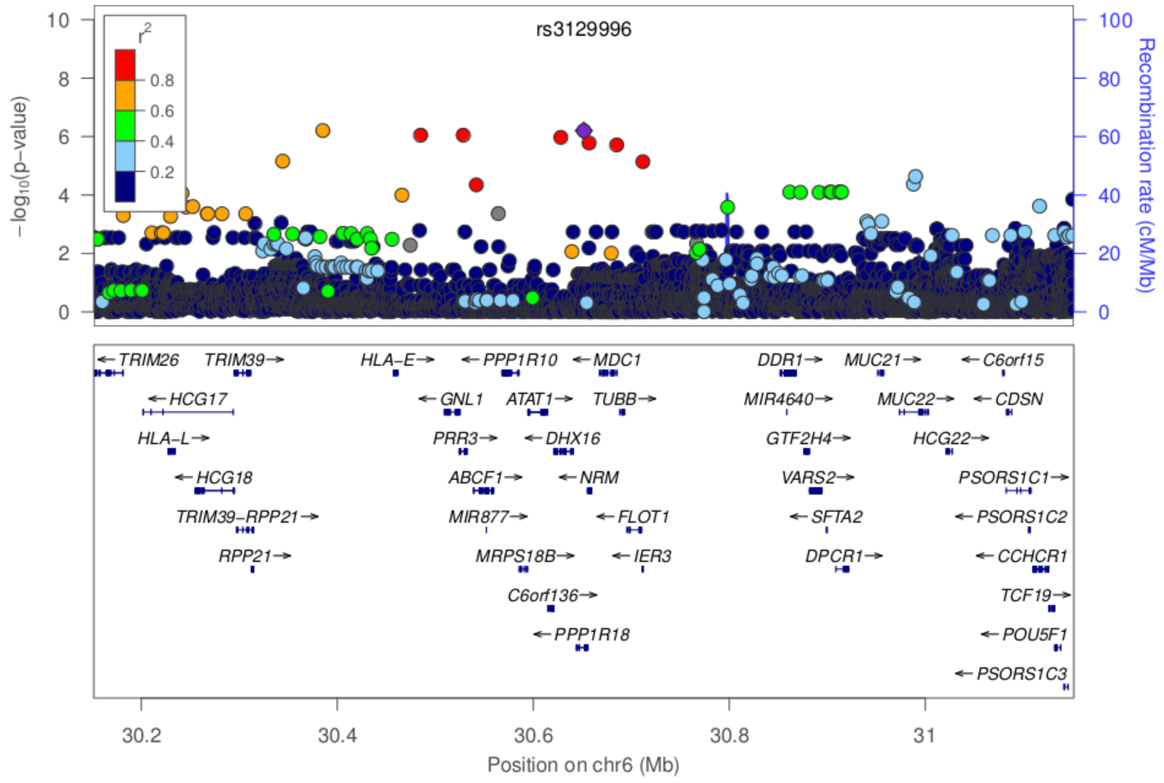

Supplement: Supplementary Informations [file tp201597x1.pdf]
